# Adjunctive corticosteroid therapy for inpatients with *Mycoplasma pneumoniae* pneumonia

**DOI:** 10.1186/s12890-017-0566-4

**Published:** 2017-12-29

**Authors:** Masato Tashiro, Kiyohide Fushimi, Kei Kawano, Takahiro Takazono, Tomomi Saijo, Kazuko Yamamoto, Shintaro Kurihara, Yoshifumi Imamura, Taiga Miyazaki, Katsunori Yanagihara, Hiroshi Mukae, Koichi Izumikawa

**Affiliations:** 1 Department of Infectious Diseases, Nagasaki University Graduate School of Biomedical Sciences, 1-7-1 Sakamoto, Nagasaki, 852-8501 Japan; 2 Nagasaki University Infection Control and Education Centre, Nagasaki University Hospital, Nagasaki, Japan; 3 Department of Health Policy and Informatics, Graduate School of Medicine, Tokyo Medical and Dental University, Tokyo, Japan; 4 Second Department of Internal Medicine, Nagasaki University Hospital, Nagasaki, Japan; 5 Department of Laboratory Medicine, Nagasaki University Hospital, Nagasaki, Japan

**Keywords:** *Mycoplasma pneumoniae*, Pneumonia, Corticosteroid

## Abstract

**Background:**

There is conflicting evidence regarding the benefit of adjunctive corticosteroid therapy in patients with *Mycoplasma pneumoniae* pneumonia. We hypothesised that corticosteroid therapy could reduce mortality and length of stay (LOS) in such patients.

**Methods:**

Adult patients with *M. pneumoniae* pneumonia from January 2010 to December 2013 were identified from the Japanese Diagnosis Procedure Combination inpatient database. The effects of low-dose and high-dose corticosteroid therapies on mortality, LOS, drug costs and hyperglycaemia requiring insulin treatment were evaluated using propensity score analyses.

**Results:**

Eligible patients (*n* = 2228) from 630 hospitals were divided into no-corticosteroid (*n* = 1829), low-dose corticosteroid (*n* = 267) and high-dose corticosteroid (*n* = 132) groups. The propensity score-matched pairs were generated from no-corticoid and low-dose corticoid groups (251 pairs), or no-corticoid and high-dose corticosteroid groups (120 pairs). Adjunctive corticosteroid therapy did not decrease 30-day mortality. In addition, both low-dose and high-dose corticosteroid therapies were associated with increases in LOS. Furthermore, hyperglycaemia requiring insulin treatment and drug cost increased with corticosteroid use.

**Conclusions:**

Adjunctive treatment with low-dose or high-dose corticosteroids may not be beneficial in *M. pneumoniae* pneumonia.

## Background

*Mycoplasma pneumoniae* is the cause of 3–10% of pneumonia cases [1] and accounts for 10–30% of all cases of community-acquired pneumonia (CAP) [2, 3]. Most cases of *M. pneumoniae* pneumonia are mild and self-limiting; however, some result in fulminant respiratory failure and may be fatal [4–8]. A previous study reported a mortality rate of 29.4% among patients with *M. pneumoniae* pneumonia who required intensive care unit (ICU) admission [9]. In addition to antimicrobial agents, corticosteroids may be beneficial in the treatment of severe cases of *M. pneumoniae* infections. This is because the pathogenesis of the disease is related to excessive immune responses, including highly activated cell-mediated immune responses and high expressions of cytokines [10–14].

Corticosteroids have been shown to exert a beneficial effect in severe cases of *M. pneumoniae* infection via downregulation of cell-mediated immune responses associated with the pulmonary injury that occurs during the infection [15–18]. However, evidence is limited to case reports and small case series. The results of studies in patients with severe CAP suggest that short-term, late-phase, high-dose corticosteroid treatment is not beneficial [19–22]. Conversely, other studies have reported the efficacy of high-dose methylprednisolone therapy in patients with severe *M. pneumoniae* pneumonia [23–26]. In the present study, we hypothesised that adjunctive corticosteroid therapy may reduce mortality and length of stay (LOS) in patients with severe *M. pneumoniae* pneumonia.

## Methods

### Data source

We used a large nationwide dataset that was obtained from the Japanese Diagnosis Procedure Combination (DPC) system. The database contains claims and abstracted discharge data from >1000 participating hospitals, including 92% (244/266) of all tertiary hospitals in Japan [27]. The baseline patient information includes age, sex, primary diagnosis and comorbidities at admission, which are coded according to the International Classification of Diseases, 10th revision (ICD-10) [28]. The database also includes the dosages of all drugs and blood products administered during hospitalisation, as well as the dates of treatment administration. In addition, all interventional procedures are coded with original Japanese codes. Dates of hospital admission and discharge, bedside procedures, drugs administered and discharge status (dead or alive) are recorded using a uniform data submission format. Moreover, diagnostic records are linked to the payment system and attending physicians are required to report objective evidence of each diagnosis made for reimbursement of treatment [27]. The DPC is an administrative database with information inputted at discharge. Thus, for the present study, patient follow-up began on the day of admission and ended on the day of discharge, transfer or death. It was impossible to follow up patients thereafter since no subsequent data were entered.

Data were anonymised on extraction and analysed within the protected environment of the Nagasaki University Hospital (Nagasaki, Japan). The Institutional Review Board of Nagasaki University Hospital waived the requirement of informed consent and approved the study design (Institutional Review Board No. 17012303).

### Patient selection

We identified patients diagnosed with *M. pneumoniae* pneumonia from January 2010 to December 2013. During this period, multiple large epidemics of *M. pneumoniae* infections occurred in Japan [29]. We included patients who were ≥18 years old and had undergone diagnostic tests (paired serologic antibody titres, antigen detection or polymerase chain reaction) confirming *M. pneumoniae* infection. We restricted the analysis to adults because severe *M. pneumoniae* infections occur more frequently in the adult and elderly populations [9]. A single antibody titre and cold agglutinins were not included in the diagnostic tests in this study.

The exclusion criteria were as follows: 1) discharge within 2 days of admission, 2) start intravenous corticosteroid therapy after day 2 of admission and 3) existence of any missing data. Low-dose corticosteroid use was defined as the starting dosages an intravenous infusion of methylprednisolone at <125 mg (or an equivalent dose of dexamethasone, hydrocortisone, prednisolone or betamethasone) and any higher dose was defined as a high dose [30].

### Variables and endpoints

Comorbidities were evaluated using the Charlson comorbidity index (CCI), which is a method used to predict mortality by classifying or weighting comorbidities [28]. The CCI includes 17 conditions with major impact on survival and is widely used by health researchers to measure case mix and the burden of a disease [31]. For instance, a higher CCI score reflects a more severe comorbidity. The pneumonia severity was evaluated using the A-DROP system, which is the modified CURB-65 scoring system proposed by The Japanese Respiratory Society [32]. It assesses Age (men ≥70 years, women ≥75 years), Dehydration (existence of a clinical sign of dehydration or blood urea nitrogen level ≥ 210 mg/L), Respiratory failure (SpO_2_ ≤ 90% or PaO_2_ ≤ 60 mmHg), Orientation disturbance (confusion), and a low blood Pressure (systolic blood pressure ≤ 90 mmHg). The scoring system stratifies patients into four severity classes (mild = 0; moderate = 1–2; severe = 3; and extremely severe = 4–5), and it has an equal ability for predicting the mortality of CAP compared to the CURB-65 scoring system [33]. We assessed medications administered and interventions performed within 2 days of admission except the endpoints.

The endpoints were all-cause 30-day mortality, in-hospital hyperglycaemia needing insulin treatment, LOS, duration of antimicrobial treatment and total costs of all the drugs used. We described cost in terms of Japanese Yen (JPY), Euro (EUR) and US Dollar (USD) (1.00 JPY = 0.008587 EUR, 1.00 JPY = 0.009877 USD).

### Statistical analysis

We separately performed one-to-one matching between the no-corticosteroid and the low-dose corticosteroid group, or the no-corticosteroid and the high-dose corticosteroid groups, based on estimated propensity scores for each patient in order to minimise the bias due to confounding variables [31, 34]. To estimate the propensity score, we fitted a logistic regression model for low-dose or high-dose corticosteroid use as a function of patient and hospital demographics, which included age, sex, CCI score, comorbidities (congestive heart failure, renal disease, liver disease, diabetes with/without chronic complications, chronic obstructive pulmonary disease, bronchial asthma, interstitial lung disease and fibrosis, pleural effusion and aspiration or drainage of pleural effusion), parameters of the A-DROP, pneumonia severity, antimicrobial drugs (macrolides, quinolones and tetracyclines), other supportive drugs (catecholamines), interventions (invasive and non-invasive ventilation) and organizational characteristics (ICU admission, academic hospital, and average number of patients in hospital per day) [6, 26, 30, 35, 36]. The catecholamines included dopamine, dobutamine and noradrenaline (norepinephrine). The c-statistic for evaluating the goodness of fit was calculated and a one-to-one matched analysis using nearest-neighbour matching was performed based on the patients’ estimated propensity scores. A match occurred when a patient in the low-dose or high-dose corticosteroid group had an estimated score within 0.2 standard deviations (SDs) of the score of a patient in the no-corticosteroid group [31]. Descriptive statistics have been presented for all patients, including the propensity score-matched patients. Fisher’s exact test or Pearson’s chi-square test was used to compare discrete variables, whereas the Wilcoxon rank-sum test or Kruskal–Wallis test was used for continuous variables. All the statistical analyses were performed using JMP 12.0 software (SAS Institute, Cary, NC, USA). All the tests were two-tailed and a *p* value <0.05 was considered statistically significant. Data have been expressed as mean ± SD.

## Results

### Patients

**Fig. 1:**
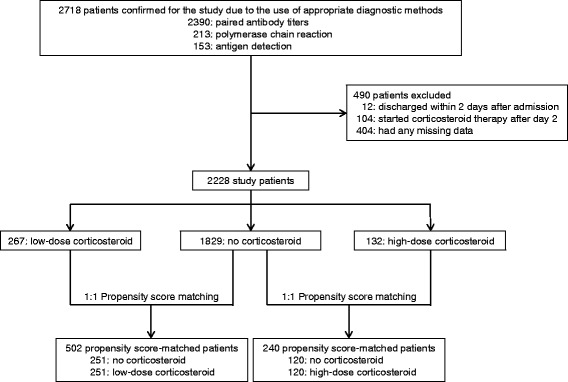
Patient selection

A total of 2718 patients with *M. pneumoniae* pneumonia were admitted to 690 hospitals from January 2010 to December 2013. Among them, 2390 patients were diagnosed by paired antibody titers, 213 by polymerase chain reaction, and 153 by antigen detection (Fig. 1). Overall, 490 patients were excluded based on the various exclusion criteria previously mentioned. As a result, 2228 patients from 630 hospitals were used in the study and were divided into the following groups: no-corticosteroid (*n* = 1829), low-dose corticosteroid (*n* = 267) and high-dose corticosteroid (*n* = 132). From the aforementioned data, the low-dose and high-dose corticosteroid propensity score-matched pairs that were generated were 247 and 122, respectively (Fig. 1). The c-statistic analysis indicated that the goodness of fit values were 0.79 and 0.83 for the low-dose and high-dose groups, respectively, according to the propensity score model.

Table 1 shows the baseline characteristics of the unmatched groups. When the unmatched groups were compared, patients who had more complex comorbidities (i.e., those who were treated with quinolones or were in serious condition and required supportive treatment, mechanical ventilation or care in an ICU) were more likely to receive a corticosteroid treatment. Especially, patients who had bronchial asthma tended to require low-dose corticosteroids (41.2%), whereas those who had interstitial lung disease tended to require high-dose corticosteroids (25.8%). After propensity score-matching, the baseline patient characteristics were similar between the no-corticosteroid (*n* = 251) and low-dose corticosteroid groups (n = 251), as well as between the no-corticosteroid (*n* = 120) and high-dose corticosteroid groups (n = 120) (Tables 2 and 3).

**Table 1:** Baseline patient and organization characteristics in the unmatched groups

Characteristic	No corticosteroid		Low-dose corticosteroids		High-dose corticosteroids		
	*n* = 1829		*n* = 267		*n* = 132		*p* value
Patient Characteristics							
Age, years	49.2	±22.6	49.8	±21.1	53.7	±22.3	0.0760
Male	847	(46.3)	106	(39.7)	69	(52.3)	0.0405
Preexisting comorbid conditions							
Charlson comorbidity index	0.63	±1.0	1.17	±1.2	0.88	±0.9	< 0.0001
Congestive heart failure	108	(5.9)	35	(13.1)	10	(7.6)	< 0.0001
Renal disease	32	(1.8)	3	(1.1)	2	(1.5)	0.7493
Liver disease	92	(5.0)	5	(1.9)	6	(4.5)	0.0717
Diabetes without chronic complications	144	(7.9)	25	(9.4)	17	(12.9)	0.1086
Diabetes with chronic complications	33	(1.8)	6	(2.2)	1	(0.8)	0.5723
Chronic obstructive pulmonary disease	85	(4.6)	22	(8.2)	8	(6.1)	0.0413
Bronchial asthma	157	(8.6)	110	(41.2)	31	(23.5)	< 0.0001
Interstitial lung disease and fibrosis	62	(3.4)	19	(7.1)	34	(25.8)	< 0.0001
Pleural effusion	117	(6.4)	14	(5.2)	11	(8.3)	0.4910
Aspiration or drainage of pleural effusion	4	(0.2)	3	(1.1)	2	(1.5)	0.0108
Pneumonia characteristics							
Parameters of the A-DROP							
Age (male ≥ 70 years, female ≥ 75 years)	473	(25.9)	58	(21.7)	43	(32.6)	0.0642
Dehydration	201	(11.0)	39	(14.6)	19	(14.4)	0.1344
Confusion	196	(10.7)	63	(23.6)	56	(42.4)	< 0.0001
Respiratory failure	38	(2.1)	12	(4.5)	8	(6.1)	0.0025
Low blood pressure	176	(9.6)	37	(13.9)	17	(12.9)	0.0638
Pneumonia severity							
Mild	1110	(60.7)	137	(51.3)	48	(36.4)	< 0.0001
Moderate	645	(35.3)	111	(41.6)	75	(56.8)	
Severe	58	(3.2)	14	(5.2)	6	(4.5)	
Extremely severe	16	(0.9)	5	(1.9)	3	(2.3)	
Other treatments							
Anti-mycoplasmal drug use							
Macroride	695	(38.0)	104	(39.0)	49	(37.1)	0.9314
Quinolone	548	(30.0)	116	(43.4)	69	(52.3)	< 0.0001
Tetracycline	569	(31.1)	78	(29.2)	30	(22.7)	0.1172
Other supportive drug use							
Catecholamine	13	(0.7)	5	(1.9)	8	(6.1)	< 0.0001
Interventions							
Invasive ventilation	10	(0.5)	5	(1.9)	12	(9.1)	< 0.0001
Non-invasive ventilation	2	(0.1)		(0.0)	2	(1.5)	0.0009
Organization characteristics							
Intensive care unit admission	8	(0.4)	5	(1.9)	10	(7.6)	< 0.0001
Academic hospital	131	(7.2)	16	(6.0)	17	(12.9)	0.0345
Average number of patients in hospital per day							
≤ 200	351	(19.2)	40	(15.0)	26	(19.7)	0.2502
201–600	1194	(65.3)	190	(71.2)	81	(61.4)	
≥ 601	284	(15.5)	37	(13.9)	25	(18.9)	

Data are shown as n (%) or mean ± SD (standard deviation)

**Table 2:** Baseline patient and organization characteristics in the matched groups of no corticosteroid and low-dose corticosteroid

Characteristic	No corticosteroid		Low-dose corticosteroids		
	*n* = 251		*n* = 251		*p* value
Patient Characteristics					
Age, years	49.9	±21.6	49.6	±21.5	0.8753
Male	110	(43.8)	101	(40.2)	0.4695
Preexisting comorbid conditions					
Charlson comorbidity index	1.25	±1.3	1.08	±1.1	0.1921
Congestive heart failure	30	(12.0)	30	(12.0)	1.0000
Renal disease	5	(2.0)	3	(1.2)	0.7244
Liver disease	6	(2.4)	5	(2.0)	1.0000
Diabetes without chronic complications	27	(10.8)	25	(10.0)	0.8837
Diabetes with chronic complications	8	(3.2)	6	(2.4)	0.7875
Chronic obstructive pulmonary disease	25	(10.0)	20	(8.0)	0.5324
Bronchial asthma	103	(41.0)	96	(38.2)	0.5841
Interstitial lung disease and fibrosis	12	(4.8)	16	(6.4)	0.5603
Pleural effusion	12	(4.8)	13	(5.2)	1.0000
Aspiration or drainage of pleural effusion	2	(0.8)	2	(0.8)	1.0000
Pneumonia characteristics					
Parameters of the A-DROP					
Age (male ≥70 years, female ≥75 years)	61	(24.3)	57	(22.7)	0.7523
Dehydration	40	(15.9)	35	(13.9)	0.6167
Confusion	47	(18.7)	54	(21.5)	0.5043
Respiratory failure	8	(3.2)	9	(3.6)	1.0000
Low blood pressure	29	(11.6)	31	(12.4)	0.8907
Pneumonia severity					
Mild	137	(54.6)	135	(53.8)	0.9527
Moderate	97	(38.6)	100	(39.8)	
Severe	13	(5.2)	11	(4.4)	
Extremely severe	4	(1.6)	5	(2.0)	
Other treatments					
Anti-mycoplasmal drug use					
Macroride	89	(35.5)	94	(37.5)	0.7107
Quinolone	104	(41.4)	106	(42.2)	0.9279
Tetracycline	68	(27.1)	75	(29.9)	0.5531
Other supportive drug use					
Catecholamine	4	(1.6)	4	(1.6)	1.0000
Interventions					
Invasive ventilation	5	(2.0)	4	(1.6)	1.0000
Non-invasive ventilation	0	(0.0)	0	(0.0)	
Organization characteristics					
Intensive care unit admission	4	(1.6)	5	(2.0)	1.0000
Academic hospital	12	(4.8)	15	(6.0)	0.6931
Average number of patients in hospital per day					
≤ 200	44	(17.5)	40	(15.9)	0.7962
201–600	169	(67.3)	176	(70.1)	
≥ 601	38	(15.1)	35	(13.9)	

Data are shown as n (%) or mean ± SD (standard deviation)

**Table 3:** Baseline patient and organization characteristics in the matched groups of no corticosteroid and high-dose corticosteroid

Characteristic	No corticosteroid		High-dose corticosteroids		
	n = 120		n = 120		*p* value
Patient Characteristics					
Age, years	55.2	±22.7	52.5	±22.5	0.3607
Male	65	(54.2)	63	(52.5)	0.8971
Preexisting comorbid conditions					
Charlson comorbidity index	1.01	±1.1	0.90	±0.9	0.6511
Congestive heart failure	11	(9.2)	9	(7.5)	0.8160
Renal disease	1	(0.8)	2	(1.7)	1.0000
Liver disease	4	(3.3)	6	(5.0)	0.7486
Diabetes without chronic complications	15	(12.5)	14	(11.7)	1.0000
Diabetes with chronic complications		(0.0)	1	(0.8)	1.0000
Chronic obstructive pulmonary disease	11	(9.2)	8	(6.7)	0.6336
Bronchial asthma	39	(32.5)	30	(25.0)	0.2538
Interstitial lung disease and fibrosis	23	(19.2)	24	(20.0)	1.0000
Pleural effusion	10	(8.3)	10	(8.3)	1.0000
Aspiration or drainage of pleural effusion	1	(0.8)	1	(0.8)	1.0000
Pneumonia characteristics					
Parameters of the A-DROP					
Age (male ≥70 years, female ≥75 years)	40	(33.3)	36	(30.0)	0.6773
Dehydration	22	(18.3)	18	(15.0)	0.6037
Confusion	43	(35.8)	45	(37.5)	0.8935
Respiratory failure	6	(5.0)	6	(5.0)	1.0000
Low blood pressure	23	(19.2)	16	(13.3)	0.2937
Pneumonia severity					
Mild	45	(37.5)	48	(40.0)	0.7779
Moderate	64	(53.3)	64	(53.3)	
Severe	5	(4.2)	5	(4.2)	
Extremely severe	6	(5.0)	3	(2.5)	
Other treatments					
Anti-mycoplasmal drug use					
Macroride	44	(36.7)	45	(37.5)	1.0000
Quinolone	49	(40.8)	59	(49.2)	0.2429
Tetracycline	28	(23.3)	28	(23.3)	1.0000
Other supportive drug use					
Catecholamine	8	(6.7)	5	(4.2)	0.5702
Interventions					
Invasive ventilation	7	(5.8)	7	(5.8)	1.0000
Non-invasive ventilation	1	(0.8)	2	(1.7)	1.0000
Organization characteristics					
Intensive care unit admission	5	(4.2)	6	(5.0)	1.0000
Academic hospital	11	(9.2)	15	(12.5)	0.5339
Average number of patients in hospital per day					
≤ 200	18	(15.0)	23	(19.2)	0.6922
201–600	79	(65.8)	75	(62.5)	
≥ 601	23	(19.2)	22	(18.3)	

Data are shown as n (%) or mean ± SD (standard deviation)

The durations of intravenous corticosteroid use were 3.8 ± 9.7 days (unmatched group) and 3.4 ± 9.4 days (matched group) in the low-dose corticosteroid group, and 2.8 ± 3.6 days (unmatched group) and 2.9 ± 3.6 days (matched group) in the high-dose corticosteroid group. The stated durations included the periods of tapered low-dose corticosteroid use. The starting dosages of methylprednisolone were 56.3 ± 39.4 mg/day (unmatched group) and 55.7 ± 39.7 mg/day (matched group) in the low-dose corticosteroid group, and 622 ± 422 mg/day (unmatched group) and 603 ± 430 mg/day (matched group) in the high-dose corticosteroid group.

### Endpoints

The outcomes in the unmatched groups showed a higher 30-day mortality rate (5.3%) in the high-dose corticosteroid group than in the no-corticosteroid group (0.8%) or low-dose corticosteroid group (1.9%) (*p* < 0.0001) (Table 4). Furthermore, the occurrence of in-hospital hyperglycaemia that required insulin treatment, which is an important adverse effect of corticosteroid therapy, was significantly higher at higher corticosteroid dosages (5.7, 11.2 and 23.5% for the no-corticosteroid, low-dose corticosteroid and high-dose corticosteroid groups, respectively). In addition, LOS significantly increased as corticosteroid dosage was increased (13.1 ± 12.8, 18.0 ± 18.8 and 23.6 ± 18.5 days for the no-corticosteroid, low-dose corticosteroid and high-dose corticosteroid groups, respectively). Moreover, the duration of antimicrobial treatment was longer in the low-dose and high-dose corticosteroid groups (9.3 ± 7.3 and 9.5 ± 6.3 days, respectively) than in the no-corticosteroid group (6.8 ± 4.8 days) (*p* < 0.001). The total cost of all drugs administered during hospitalisation was higher in the corticosteroid-treated groups than in the no-corticosteroid group (*p* < 0.0001).

**Table 4:** Comparisons of outcomes between unmatched groups

Characteristic	No corticosteroid		Low-dose corticosteroids		High-dose corticosteroids		
	*n* = 1829		*n* = 267		*n* = 132		*p* value
30-day mortality	14	(0.8)	5	(1.9)	7	(5.3)	< 0.0001
In-hospital hyperglycemia requiring insulin treatment	104	(5.7)	30	(11.2)	31	(23.5)	< 0.0001
Length of stay, days	13.1	±12.8	18.0	±18.8	23.6	±18.5	< 0.0001
Length of anitmicrobial treatment, days	6.8	±4.8	9.3	±7.3	9.5	±6.3	< 0.0001
Total cost of all drugs in hospitalization							
Japanese Yen ± SD	49,799	±99,142	107,656	±243,792	161,980	±225,494	< 0.0001
Euro ± SD	428	±851	924	±2093	1391	±1936	
US Dollar ± SD	492	±979	1063	±2408	1600	±2227	

Data are shown as n (%) or mean ± SD (standard deviation)

1.00 Japanese Yen = 0.008587 Euro

1.00 Japanese Yen = 0.009877 US Dollar

In the propensity score-matched groups, low-dose corticosteroid therapy was associated with a long LOS, a long antimicrobial treatment, and a high total cost of drug treatment (Table 5). There were no statistical differences in 30-day mortality and hyperglycaemia requiring insulin treatment in the propensity score analysis between the no-corticosteroid and low-dose corticosteroid groups.

**Table 5:** Comparisons of outcomes between matched groups of no corticosteroid and low-dose corticosteroid

Characteristic	No corticosteroid		Low-dose corticosteroids		
	*n* = 251		*n* = 251		*p* value
30-day mortality	5	(2.0)	5	(2.0)	1.0000
In-hospital hyperglycemia requiring insulin treatment	20	(8.0)	30	(12.0)	0.1794
Length of stay, days	12.8	±9.1	17.6	±18.0	0.0055
Length of anitmicrobial treatment, days	7.1	±5.1	8.9	±6.7	0.0026
Total cost of all drugs in hospitalization					
Japanese Yen ± SD	63,265	±142,385	103,566	±237,757	< 0.0001
Euro ± SD	543	±1223	889	±2042	
US Dollar ± SD	625	±1406	1023	±2348	

Data are shown as n (%) or mean ± SD (standard deviation)

1.00 Japanese Yen = 0.008587 Euro

1.00 Japanese Yen = 0.009877 US Dollar

The high-dose corticosteroid group also showed a long LOS, a long antimicrobial treatment and a high total cost of drug treatment (Table 6). There was no statistical difference in 30-day mortality between the no-corticosteroid and high-dose corticosteroid groups. However, an extremely high prevalence of hyperglycaemia requiring insulin treatment was seen in the high-dose corticosteroid group (5.0% [no-corticosteroid] vs 21.7% [high-dose corticosteroid], *p* = 0.0002).

**Table 6:** Comparisons of outcomes between matched groups of no corticosteroid and high-dose corticosteroid

Characteristic	No corticosteroid		High-dose corticosteroids		
	*n* = 122		*n* = 122		*p* value
30-day mortality	4	(3.3)	6	(5.0)	0.7486
In-hospital hyperglycemia requiring insulin treatment	6	(5.0)	26	(21.7)	0.0002
Length of stay, days	15.4	±17.8	22.8	±18.2	< 0.0001
Length of anitmicrobial treatment, days	6.9	±5.2	9.4	±6.4	0.0005
Total cost of all drugs in hospitalization					
Japanese Yen ± SD	94,905	±222,024	145,767	±203,214	< 0.0001
Euro ± SD	815	±1907	1252	±1745	
US Dollar ± SD	937	±2193	1440	±2007	

Data are shown as n (%) or mean ± SD (standard deviation)

1.00 Japanese Yen = 0.008587 Euro

1.00 Japanese Yen = 0.009877 US Dollar

## Discussion

Based on our hypothesis, our aim was to demonstrate the beneficial effects of adjunctive corticosteroid therapy for *M. pneumoniae* pneumonia. Before conducting this analysis, we had expected that this adjunct therapy could rapidly improve the systemic symptoms of patients and shorten hospitalisation and decrease total treatment costs. However, contrary to our hypothesis, we found that adjunctive treatment with either low-dose or high-dose corticosteroids was associated with a longer LOS and a higher cost of drug treatment during hospitalisation. This means that patients who were administered corticosteroids were not discharged earlier than those who were not administered corticosteroids were. Moreover, a longer LOS is likely to result in the administration of more drugs, which increases the cost of pharmacological treatment. Furthermore, physicians administered corticosteroids as an adjunct therapy in severe cases or to patients who had bronchial asthma or interstitial lung disease. Indeed, from the unmatched group analysis, we found that disease severity was significantly greater in the corticosteroid groups than in the no-corticosteroid group, which clearly influenced mortality, LOS and other outcomes. Hence, we performed a propensity-matched analysis to balance out the outcomes of disease severity. LOS and total drug cost were still higher in the corticosteroid groups than in the no-corticosteroid group. We observed that adjunctive corticosteroid therapy could not reduce LOS or total drug costs although clinicians have observed clinical improvements immediately after administering corticosteroids in some cases [25]. Our results do not necessarily conflict with the outcomes stated in the aforementioned report, which indicated early beneficial effects of adjunctive corticosteroid treatment. This is because later outcomes such as LOS and total drug costs were not stated in the report. In addition, we did not observe a decrease in mortality in the corticosteroid-treated patients, which is a difficult outcome to assess because of the low mortality rate from *M. pneumoniae* infections.

Recent randomised controlled trials (RCTs) and cohort studies that evaluated the use of corticosteroids in adult patients with CAP showed the possibility of obtaining beneficial effects from the treatment, such as decreased treatment failure and shortened time to achieve clinical stability [35, 36]. This call for the use of steroids during the treatment of patients with pneumonia is relevant since *M. pneumoniae* is one of the two most common respiratory pathogens [35]. However, the results from our study do not corroborate those of some previous studies. Torres et al. showed that acute administration of methylprednisolone for patients with severe community-acquired pneumonia and high initial inflammatory response was associated with few treatment failures and low inflammatory responses [35]. Our study included pneumonia patients regardless of the degree of inflammation because we could not use C-reactive protein which is a marker of the inflammation despite we tried to adjust pneumonia severity by using A-DROP system. These differences of results between previous studies and our study indicate that adjunctive steroid therapy could be beneficial in particular cases and that inadequate steroid administration might lead to unfavourable effects.

The main strength and the most important characteristic of our study, which makes it unique from previous studies, is that we analysed the effects of corticosteroid therapy in three groups of patients. Recent studies have focused on only low-dose corticosteroid therapy; however, the efficacy of high-dose methylprednisolone therapy has also been reported for the treatment of severe *M. pneumoniae* pneumonia [23–26]. Therefore, we analysed the efficacy of corticosteroids as an adjunctive therapy at both low and high doses. However, our findings indicate that high-dose corticosteroid therapy might induce several adverse effects, including hyperglycaemia, which might result in a longer LOS and a higher drug cost due to the need to manage the adverse effects with drugs. These results might apply to the treatment of CAP as well.

This study has some limitations. Firstly, many of the diagnoses of *M. pneumoniae* pneumonia were made based on paired antibody titres. Therefore patients with longer LOS were selected for this analysis, which could be considered as selection bias. Indeed, the mean LOS in this study was longer than 2 weeks. However, the abovementioned limitation affected the no-corticosteroid and corticosteroid-treated groups in similar manners. Secondly, although we used a nationwide database, our study was retrospective, observational and conducted without randomisation. Moreover, bias arising from unmeasured confounders, such as clinical variables and laboratory values, may have been present although we used propensity score matching to adjust for differences in baseline characteristics and disease severity [21, 35]. Therefore, large randomised trials are necessary to confirm these results; however, it may not be easy to perform such trials considering that life-threatening *M. pneumoniae* pneumonia is a rare condition. Thus, the present study may provide the best attainable level of evidence on the issue under discussion. Thirdly, there is a possibility that the study population included some patients with bacterial pneumonia caused by other bacteria in addition to *M. pneumoniae*. However, in routine clinical settings it is difficult to exclude co-infection with other pathologic bacteria in patients diagnosed with severe *M. pneumoniae* pneumonia.

## Conclusions

We found that adjunctive treatment with either low-dose or high-dose corticosteroids may not be beneficial in patients with *M. pneumoniae* pneumonia. Based on these findings, clinicians should be more careful if they use corticosteroids to treat adult inpatients with *M. pneumoniae* pneumonia.

## Abbreviations

CCI: Charlson comorbidity index
CI: Confidence interval
DPC: Japanese Diagnosis Procedure Combination
ICD-10: International Classification of Diseases, tenth revision codes
JCS: Japan Coma Scale
LOS: Length of stay
OR: Odds ratio
SD: Standard deviation
